# The use of prophylactic anticonvulsants in patients with brain tumours—a systematic review

**DOI:** 10.3747/co.v13i6.107

**Published:** 2006-12

**Authors:** J. Perry, L. Zinman, A. Chambers, K. Spithoff, N. Lloyd, N. Laperriere, on behalf of the Neuro-oncology Disease Site Group, of Cancer Care Ontario’s Program in Evidence-Based Care

**Affiliations:** * Toronto–Sunnybrook Regional Cancer Centre, Toronto, Ontario; † Cancer Care Ontario, Program in Evidence-Based Care, McMaster University, Hamilton, Ontario.; ‡ Princess Margaret Hospital, Toronto, Ontario

**Keywords:** Brain tumours, seizures, anticonvulsants, systematic review

## Abstract

**Questions:**

Should patients with newly diagnosed brain tumours receive prophylactic anticonvulsants to reduce seizure risk?

What is the best practice for patients with brain tumours who are taking anticonvulsant medications but who have never had a seizure?

**Perspectives:**

Patients with primary or metastatic brain tumours who have never had a seizure still have a 20% risk of experiencing a seizure over the course of their disease. Because considerable practice variation exists in regard to the management of patients with brain tumours who have never had a seizure, and because conflicting evidence has been reported, the Neuro-oncology Disease Site Group (dsg) of Cancer Care Ontario’s Program in Evidence-based Care felt that a systematic review of the evidence was warranted.

**Outcomes:**

Outcomes of interest were incidence of seizures and adverse effects of prophylactic anticonvulsant therapy.

**Methodology:**

The medline and Cochrane Library databases were systematically searched for relevant evidence. The review included fully published reports or abstracts of randomized controlled trials (rcts), systematic reviews, meta-analyses, and practice guidelines.

The present systematic review was reviewed and approved by the Neuro-oncology dsg, which comprises medical and radiation oncologists, surgeons, neurologists, a nurse, and a patient representative.

**Results:**

**Quality of Evidence**

The literature search located one evidence-based practice guideline, one systematic review, and five rcts that addressed prophylactic anticonvulsants for patients with brain tumours. Evidence for the best management of seizure-naïve patients who are already taking anticonvulsants was limited to one retrospective study and exploratory analyses within several rcts.

**Benefits and Harms**

Pooled results of the five rcts suggest that the incidence of seizures in patients who receive prophylactic anticonvulsants is not significantly different from that in patients who do not receive anticonvulsants (relative risk: 1.04; 95% confidence interval: 0.70 to 1.54; *p* = 0.84). This analysis accords with results from a published meta-analysis.

Evidence is insufficient to determine whether patients who are currently taking anticonvulsants but who have never had a seizure should taper the anticonvulsants. Patients who received anticonvulsants reported adverse effects, including rash, nausea, and hypotension, but whether these effects are a result of the anticonvulsants or of other treatments could not be determined.

**Conclusions:**

Based on the available evidence, the routine use of postoperative anticonvulsants is not recommended in seizure-naïve patients with newly diagnosed primary or secondary brain tumours, especially in light of a significant risk of serious adverse effects and problematic drug interactions. Because data are insufficient to recommend whether anticonvulsants should be tapered in patients who are already taking anticonvulsants but who have never had a seizure, treatment must be individualized.

## 1. QUESTIONS

Should patients with newly diagnosed brain tumours receive prophylactic anticonvulsants to reduce seizure risk?

What is the best practice for patients with brain tumours who are taking anticonvulsant medications but who have never had a seizure?

## 2. CHOICE OF TOPIC AND RATIONALE

Approximately 25% of patients with newly diagnosed primary or secondary brain tumours have seizures as a presenting symptom. Seizures may be more common in patients with low-grade infiltrative tumours, tumours near the motor cortex, and hemorrhagic tumours 1. If seizures have not occurred at presentation, a 20% risk of having a seizure at some point during the course of the disease remains.

Seizures are an important determinant of quality of life in these patients. Seizures threaten independence, may cause injury or loss of motor function, may necessitate hospitalization, and increase the need for higher-dose or additional anticonvulsants, with increased adverse effects. Even in patients with no active seizures, the fear of seizures affects patient well-being and increases caregiver stress.

Best practices for the appropriate use of anticonvulsants in these patients have not been established. Clearly, there is a role for anticonvulsants in patients with known seizures and in craniotomy patients in general as prophylaxis during the perioperative period, but the role of long-term prophylactic anticonvulsants for patients without a history of seizures is not as clear. Furthermore, practice varies considerably in the management of patients who are prescribed anticonvulsants during the perioperative period and who then remain on this treatment during follow-up.

Before commencing development of this systematic review, the Neuro-oncology Disease Site Group (dsg) of Cancer Care Ontario’s Program in Evidence-Based Care (pebc) surveyed 197 practitioners in Ontario regarding their current practices when prescribing anticonvulsants to patients with newly diagnosed brain tumours 2. A total of 197 surveys were sent to medical oncologists, neurologists, radiation oncologists, and surgeons, and 125 practitioners (63%) responded.

The survey included three scenarios addressing the use of anticonvulsants in common clinical situations: perioperatively, in patients without seizures; postoperatively, in patients currently using anticonvulsants; and in patients not currently using anticonvulsants and not undergoing surgery. The first two situations yielded considerable variation in practitioner response; the final scenario, less variation. It is important to recognize that variations in practice do not necessarily imply an explicitly wrong or right practice. Such variations can be based on many factors, including patients’ needs, morbidity rates, and variations in consumer preferences for particular outcomes 3. However, because of these differences in patient management, the Neuro-oncology dsg felt that a systematic review of the evidence was warranted as the basis for a practice guideline to be disseminated to Ontario practitioners.

## 3. METHODS

The present systematic review was originally completed in the context of developing an evidence-based series, including a clinical practice guideline, using the methodology of the practice guidelines development cycle 4. The evidence was selected and reviewed by members of the Neuro-oncology dsg and by methodologists. The pebc is editorially independent of Cancer Care Ontario and the Ontario Ministry of Health and Long-Term Care. Evidence-based reports produced by the pebc undergo periodic review, and new evidence is incorporated into the original reports as appropriate. The most recent versions of these reports can be found at the Cancer Care Ontario Web site (www.cancercare.on.ca/index_practiceGuidelines.htm).

### 3.1 Literature Search Strategy

A systematic search was conducted of the medline (1966 to June 2005) and Cochrane Library (Issue 2, 2005) databases using “anticonvulsant” [Medical Subject Heading [mesh]) or “antiepileptic drugs” (mesh) combined with the keywords “glioma,” “glioblastoma,” and “brain tumours.” These terms were then combined with the search terms for the following study designs: practice guidelines, systematic reviews, meta-analyses, reviews, randomized controlled trials (rcts), controlled clinical trials, and retrospective studies. The Canadian Medical Association Infobase (www.cma.ca/cpgs/index.asp), the National Guidelines Clearing-house (www.guideline.gov), and other Web sites were also searched for existing evidence-based practice guidelines. Relevant articles and abstracts were selected and reviewed by three reviewers, and the reference lists from those sources were searched for additional trials, as were the reference lists from relevant review articles.

### 3.2 Study Selection Criteria

Fully published articles or abstracts were selected for inclusion in this systematic review if they

- were rcts, systematic reviews, or meta-analyses of rcts that compared patients with brain tumours treated with prophylactic anticonvulsants with patients with brain tumours not treated with prophylactic anticonvulsants, or that compared various anticonvulsant-tapering strategies in patients with brain tumours. Sufficient follow-up time was required. If no rcts were available, non-randomized studies and retrospective studies were included.
- included patients without a history of seizures.
- reported data about the incidence of seizures or adverse effects for each intervention group.
- were clinical practice guidelines from other guideline development groups evaluating the use of prophylactic anticonvulsants in patients with brain tumours.

Articles were excluded from this systematic review if they were

- publications in a language other than English.
- letters and editorials.

### 3.3 Synthesizing the Evidence

To estimate the overall effect of prophylactic anticonvulsants in patients treated with or without anticonvulsants, the incidence of seizures (the number of patients who suffered from at least one seizure by the end of the study and the number of patients included in the analysis by the investigators) was abstracted from the published reports of individual rcts. The study results were pooled using Review Manager 4.2.7 (RevMan Analyses 1.0.2, version date May 2004), which is freely available through The Cochrane Collaboration (Oxford, U.K.).

Combining data in this manner assumes a constant hazard ratio of risk for the groups being compared. Results are expressed as relative risk (rr, also known as “risk ratio”) with a 95% confidence interval (ci), where an rr less than 1 for incidence of seizures indicates fewer seizures in the experimental group. Conversely, an rr greater than 1 suggests that patients in the control group experienced fewer seizures. The rr is calculated using the ratio of the proportion of patients in the experimental treatment group who had a seizure to the proportion of patients in the control group who had a seizure. The random-effects model, being the more conservative estimate of effect, was used in preference to the fixed-effects model for pooling across studies 5.

## 4. RESULTS

### 4.1 Literature Search Results

One published evidence-based practice guideline 6 was identified for inclusion in this systematic review. Five rcts that compared anticonvulsant use to no anticonvulsant use in adults with brain tumours were also included 7–11. One published systematic review (with meta-analysis) that examined the incidence of first seizures in patients with brain tumours taking anticonvulsants was identified 12. Finally, one retrospective review examining seizure incidence in patients who discontinued anticonvulsants was also included 13.

### 4.2 Study Characteristics and Quality

All five rcts 7–11 included in this systematic review compared anticonvulsant use to no-anticonvulsant use in adults with newly diagnosed brain tumours; however, the studies used variable inclusion criteria (Table I). Two rcts were terminated early 9,11, after it was concluded that patient enrolment sufficient to detect a significant difference between treatment groups would not be feasible.

Patients in the treatment arms received phenytoin in three rcts^7^,^10^,^11^, phenytoin or phenobarbital in one RCT 8, and divalproex sodium in one rct 9. One rct examined the efficacy of anticonvulsants in the perioperative period and followed patients for 7 days 10; the median length of follow-up in the other four rcts ranged from 5.44 months to 12 months^7^–^9^,^11^. In two rcts, all patients underwent surgical resection or biopsy 7,8; in another two rcts, only some patients underwent a neurosurgical procedure 9,11.

All five rcts were fully published. The randomization method was adequately described in two trials 10,11 and was not reported in three trials 7–9. Patients were stratified by the presence of primary brain tumour or brain metastases in one rct 11. In four trials 7–10, patient stratification was not reported. Two rcts were double-blind to treatment and placebo-controlled 7,9; three rcts were open trials 8,10,11. The statistical basis for estimation of sample size and trial power was reported in three s rct 9–11.

### 4.3 Should Patients with Newly Diagnosed Brain Tumours Receive Prophylactic Anticonvulsants to Reduce Seizure Risk?

#### 4.3.1 Randomized Controlled Trials

##### Incidence of Seizures

Four rcts investigated the long-term efficacy of prophylactic anticonvulsants in patients with brain tumours who had never had a seizure 7–9,11. Two of the rcts were terminated early at the time of interim analysis, and not all patients included in these two trials underwent a neurosurgical procedure at the time of anticonvulsant administration 9,11.

The study by Forsyth *et al.* 11 was an open trial that randomized patients to receive phenytoin or no anticonvulsant therapy. The study by Glantz *et al.* 9 was double-blind, and it randomized patients to receive divalproex sodium or placebo. Forsyth *et al.* 11 permitted the inclusion of patients who had previously received anticonvulsants, but anticonvulsants were tapered in the control group before they entered the study. The study by Forsyth *et al.* 11 was terminated after 100 patients had been enrolled, and the study by Glantz *et al.* 9 was terminated when 74 patients had been enrolled.

At interim analysis, Forsyth *et al.* 11 detected no difference in seizure frequency between the two groups and noted that the incidence of seizures in the control arm was half the expected rate of 20%. Thus, the statistical power of the trial was low, and a risk reduction of 46% for seizures was ruled out. The rct reported by Glantz *et al.* 9 was designed to accrue 170 patients. Of the 37 patients in the treatment arm when the study was discontinued, 13 (35%) had had seizures, and of the 37 in the control arm, 9 (24%) had had seizures (*p* = 0.3, Table II). At the time of analysis, it was concluded that the study had reliably ruled out a difference of at least 33% in seizure incidence between the two arms. When data from that study were pooled for the purposes of the American Academy of Neurology (aan) practice parameter, Glantz *et al.* 9 concluded that the statistical power of the pooled data ruled out a risk reduction of 26% in seizure-free survival.

Two rcts examined the efficacy of anticonvulsants in patients with brain tumours who were undergoing surgical resection or biopsy 7,8. Both trials followed patients for 12 months. The study by Franceschetti *et al.* 8 was an open trial; the study by North *et al.* 7 was double-blind and placebo-controlled. Franceschetti *et al.* 8 included patients with a history of seizures, but analyzed patients without seizures separately. Only the latter results are included in the present systematic review. The relevant 63 patients were randomized to receive either anticonvulsants (phenobarbital or phenytoin) or no anticonvulsants. North *et al.* 7 included patients undergoing surgery for a variety of diagnoses, including brain tumours, but reported the results for patients with brain tumours separately. The latter 81 patients were randomized to receive either phenytoin or placebo.

Franceschetti *et al.* 8 reported 6 seizures in the anticonvulsant group (3 within 7 days of surgery) and 7 seizures in the control group (4 within 7 days of surgery). In the trial by North *et al.* 7, 9 seizures were observed in the treatment group, and 5 seizures were observed in the control group. The authors of both trials concluded that the prophylactic use of anticonvulsants was not beneficial in patients with brain tumours.

One rct was a short-term study of perioperative prophylactic anticonvulsants in which patients were followed for only 7 days 10. De Santis *et al.* randomized 200 patients undergoing surgery for brain tumours to receive either phenytoin or no add-on anticonvulsant treatment perioperatively. Most of the patients were already being treated with some form of anticonvulsant (either phenobarbital or carbamazepine) at the time of randomization (90 patients in the treatment group and 95 patients in the control group). Preoperatively, 35 patients in the treatment group and 32 patients in the control group had a history of seizures. De Santis *et al.* 10 reported that, during the 7-day observation period, 13 patients in the treatment group and 11 patients in the control group had seizures (*p* > 0.05). It is difficult to draw conclusions from this trial about the ability of anticonvulsant medication to prevent seizures because 95% of the patients in the control group were already using anticonvulsant medications.

##### Prognostic Factors

Two rcts analyzed prognostic factors for seizure occurrence 9,11. Glantz *et al.* 9 investigated tumour type, tumour location, number of lesions, age, Karnofsky performance status (kps), and extent of surgery, and concluded that none of those factors were predictive of seizure incidence. Forsyth *et al.* 11 investigated age, kps, tumour location, sex, and extent of surgical resection, and identified sex as the only prognostic factor for seizures. Women were 2.6 times more likely than were men to suffer from a seizure (95% ci: 1.01 to 6.71). No hypothesis was offered to explain that risk, nor was the number of women that experienced seizures in each group indicated. Notably, more men than women were enrolled in the study (61 men vs. 39 women); thus, the risk estimation may not be statistically robust.

##### Compliance

Four rcts reported anticonvulsant compliance 7,9–11. Forsyth *et al.* 11 measured compliance through self-reports and serum anticonvulsant levels. According to the self-reports, 93% of the patients in the anticonvulsant arm took their medication according to the prescribed schedule. Serum anticonvulsant levels were measured on two separate occasions, and 53% of the patients showed subtherapeutic levels. De Santis *et al.* 10 reported a 65%–85% compliance rate, as compared with 81% reported by North *et al.* 7, all measured by serum anticonvulsant levels. Glantz *et al.* 9 looked at compliance using pill counts and measurement of anticonvulsant serum levels. They reported that, for all patients receiving anticonvulsants, pill counts were within 5% of that expected. After 1 month, 68% of the patients had serum levels within the appropriate therapeutic range according to the study’s protocol.

##### Adverse Effects

Four rcts 7,9–11 reported adverse effects associated with anticonvulsant use. Forsyth *et al.* 11 acknowledged the difficulty of attributing adverse effects solely to the anticonvulsants, because patients were also receiving other treatments (such as chemotherapy and radiation) that are also associated with several adverse effects.

In the trial by Glantz *et al.* 9, 3 patients (8%) developed rash—2 in the divalproex sodium arm and 1 in the placebo arm. No patients withdrew from the study because of adverse effects.

In the trial by North *et al.* 7, 12 patients withdrew from the phenytoin group; rash was cited as the reason in 8 patients and involuntary movements, hirsutism, headache, and discomfort of the face were cited in 1 patient each. In the placebo arm of that trial, 3 patients withdrew because of rash, dizziness, or nausea. However, it was not reported whether the patients who withdrew had brain tumours or whether they had undergone craniotomy for other reasons.

Several adverse effects were reported in the Forsyth *et al.* 11 trial, including nausea in 4 patients (9%), rash in 3 patients (7%), and tremors, sore gums, myelosuppression, and vertigo and blurred vision in 1 patient (2%) each.

In the De Santis *et al.* 10 trial, 13 patients (13%) developed hypotension, 3 cases being severe. In addition, 3 patients (3%) experienced a mild alteration in their level of consciousness.

#### 4.3.2 Pooled Analyses of RCTs

##### Neuro-oncology dsg Meta-analysis

**FIGURE 1:**
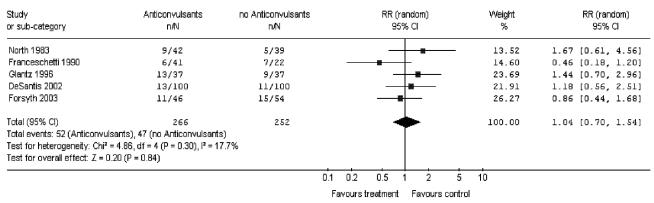
Pooled analysis of the randomized controlled trials: incidence of seizures. n = number of seizures; N = number of patients; rr = relative risk; 95% ci = 95% confidence interval.

Data were pooled from the five rcts 7–11 that compared the use of anticonvulsants to no anticonvulsants in patients with brain tumours (Figure 1). When the studies were pooled, no benefit or harm from anticonvulsants was detected in terms of the incidence of seizures (rr: 1.04; 95% ci: 0.70 to 1.54; *p* = 0.84). No significant statistical heterogeneity was observed between studies (*p* = 0.30).

##### Published Meta-analysis

Sirven *et al.* 12 published a meta-analysis evaluating studies comparing prophylactic anticonvulsant treatment to placebo or to no anticonvulsant treatment in patients with brain tumours and no history of seizures. The analysis included five trials with a total of 403 patients 7–9,11,14. The trial by Lee *et al.* 14 that was included in the meta-analysis was not included in the present systematic review because of the small number of patients with brain tumours and the short length of follow-up.

Pooling of the data showed no benefit for prophylactic anticonvulsant treatment for early-onset seizures within 1 week of treatment initiation [odds ratio (or): 0.9; 95% ci: 0.45 to 1.83; *p >* 0.05]. Pooling of the four trials with sufficient follow-up 7–9,11 for analysis of the long-term efficacy of prophylactic anticonvulsant treatment also showed no benefit (or: 1.01; 95% ci: 0.51 to 1.98; *p* > 0.05). A subgroup analysis of patients with primary glial tumours, cerebral metastases, and meningiomas showed no significant benefit for prophylactic anticonvulsant treatment in those tumour types. The authors concluded that there is little evidence to support the use of anticonvulsant prophylaxis in adult patients with brain tumours and no history of seizures.

#### 4.3.3 Published Practice Guideline

A practice parameter by the aan Practice Parameters Group lead by Glantz *et al.* 6 addressed the role of anticonvulsant prophylaxis in patients with newly diagnosed brain tumours. The practice parameter included three published rcts 7–9, one rct in press by Forsyth *et al.,* and cohort studies that investigated the role of anticonvulsants in adults with newly diagnosed brain tumours. The rct by Forsyth *et al.* was available as a pre-published manuscript, but it was never published in the journal cited. The article was published in full many years later, however 11.

Glantz *et al.* 6 conducted a meta-analysis of the four rcts and concluded that prophylactic anticonvulsants did not meaningfully reduce the risk of seizures in seizure-naïve patients with newly diagnosed brain tumours. These authors also noted that adverse effects associated with anticonvulsants were a particular concern in the given patient population. They recommended against the routine use of anticonvulsants for primary prophylaxis in newly diagnosed patients with brain tumours or brain metastases 6.

### 4.4 What Is the Best Practice for Patients with Brain Tumours Who Are Taking Anticonvulsant Medications but Who Have Never Had a Seizure?

#### 4.4.1 Randomized Controlled Trials

In the rct by North *et al.* 7, patients who were seizure-free after 12 months discontinued anticonvulsants and were followed for an additional 12 months. During this period, a first seizure occurred in 11 patients who had received anticonvulsants and in 7 patients who had received placebo. However, the trial was not designed to make that comparison, and it is not reported how many of those patients had brain tumours.

Two of the rcts included patients who were already taking anticonvulsants 10,11. Patients in those studies were randomized to receive additional anticonvulsants, to maintain their current dosage of anticonvulsants, or to taper anticonvulsants before entering the study. Neither study detected a difference in the incidence of seizures between the treatment groups, and neither study attempted to taper the use of anticonvulsants postoperatively.

#### 4.4.2 Retrospective Data

A retrospective study by Telfeian *et al.* 13 examined the results of discontinuing anticonvulsant use in 72 patients undergoing surgical resection for glioblastoma multiforme. All patients received anticonvulsants upon diagnosis, and patients who were seizure-free 6 months postoperatively had their anticonvulsants tapered and discontinued. Of 7 patients who experienced postoperative seizures, 4 were in the group of patients in whom anticonvulsants had been discontinued. Those results are consistent with the incidence of seizures expected in patients with epilepsy who have their anticonvulsants withdrawn after 2 or more years of freedom from seizure.

#### 4.4.3 Published Practice Guideline

The aan practice parameter 6 addressed the common clinical scenario of patients without a history of seizures who are prescribed prophylactic perioperative anticonvulsants. The practice parameter recommended that, “in patients with brain tumours who have not had a seizure, tapering and discontinuing anticonvulsants after the first postoperative week is appropriate, particularly in those patients who are medically stable and who are experiencing anticonvulsant-related side effects.” The authors indicated that this recommendation was based either on evidence from one or more well-designed observational studies or on expert opinion, case reports, and retrospective reviews. Glantz *et al.* did not specify the evidence on which their recommendation was based. Since the publication of the aan practice parameter, the trial by Forsyth et al. 11 has been published in full.

## 5. DISCUSSION

Only five rcts have tested the effects of anticonvulsants for the primary prophylaxis of seizures in patients with newly diagnosed brain tumours. No studies have been disease-specific, and all included a mixture of both primary and secondary brain tumours. All trials were heterogeneous with respect to inclusion criteria and the anticonvulsants used.

Anticonvulsants are problematic in patients with brain tumour. The studies of Glantz *et al.* 9 and Forsyth *et al.* 11 demonstrated that the rate and intensity of anticonvulsant-related side effects may be higher in patients with brain tumour than in patients with a seizure disorder arising from other causes. In addition, the anticonvulsants tested in those rcts were in the class of enzyme-inducing anticonvulsants. Those agents are expected to have pharmaco-dynamic interactions with other medications commonly used in the treatment of patients with brain tumour. In particular, interactions between enzyme-inducing anticonvulsants and chemotherapy are of significant concern. No studies have tested the newer generation of anticonvulsants that are, in general, characterized by fewer adverse effects and minimal drug interactions.

Because the available clinical trials were all small, it was not possible to determine if special subgroups of patients are at an increased risk of seizure. Intuitively, it might be expected that patients with tumours near the motor strip, with cortically based tumours, or with hemorrhagic tumours are at an increased risk of seizure, but no available data support this assumption. Clinicians must, therefore, individualize treatment for those patients. To reach treatment decisions, tumour-related factors such as location must be integrated with patient preferences and quality of life, plus concomitant medications.

The larger rcts and the meta-analysis demonstrate that conventional anticonvulsants (phenytoin, valproic acid, and phenobarbital) confer, at best, a 25% reduction in the risk of seizures, with incremental risk of adverse effects and drug interactions. The Neuro-oncology dsg felt that the statistical power of those trials reliably excluded a clinically important reduction in seizure risk for seizure-naïve patients with newly diagnosed primary and secondary brain tumours. Thus, the routine use of postoperative anticonvulsants is not recommended in those patients, especially in light of a significant risk of serious adverse effects and problematic drug interactions. This recommendation accords with the aan practice parameter 6.

In the survey of Ontario practitioners 2, 74% of respondents reported that they did not recommend the routine use of anticonvulsants in seizure-naïve patients with newly diagnosed brain tumours, indicating that current practice is, in the main, consistent with the dsg’s recommendation.

The newer antiepileptics may overcome some of these issues, but these agents have not been tested in clinical trials, and no recommendations can be made regarding their use. However, an rct informed by the analysis and suggestions of Forsyth *et al.* 11 should be considered.

For patients who are already on anticonvulsants but who have never had a seizure, little evidence is available to guide treatment. The aan practice parameter recommends considering tapering and discontinuation of anticonvulsants, but only one small retrospective clinical trial has attempted to address that issue. Evidence is insufficient to recommend for or against the tapering of anticonvulsants in this situation, and therefore treatment must be individualized. In the Ontario practice survey 2, essentially equal numbers of practitioners would maintain anticonvulsants, withdraw them, or have a discussion and allow a patient-based decision. Thus, current practice appears to reflect the lack of data addressing this very specific question.

## Figures and Tables

**TABLE I:** Characteristics and eligibility criteria for patients entered into the randomized controlled trials included in the systematic review

*Reference*	*Diagnosis*	*Patients (*n*)*^a^	*History of seizures*	*Anticonvulsant use*	*Other*
North *et al.*, 1983 7	Glioma	32	No prior seizures	No previous use allowed	na
	Metastases	13			
	Aneurysm	55			
	Head injury	100			
Franceschetti *et al.*, 1990 8	Glioma	55	No prior seizures	nr	na
	Metastases	19			
	Meningioma	54			
Glantz *et al.*, 1996 9	Glioma	9	No prior seizures	No previous use allowed	>18 Years
	Metastases	65			kps>50
De Santis *et al.*, 2002 10	Glioma	95	No seizures <7 days before surgery	Current use is acceptable	15–75 Years
	Metastases	10			No history of alcohol/drug abuse
	Meningioma	81			Not pregnant
	Other	14			No mi 3 months prior to surgery
Forsyth *et al.*, 2003 11	Glioma	40	No prior seizures	Current use is acceptable	Life expectancy >4 weeks
	Metastases	60			No current abuse of alcohol/drugs
					Not pregnant

a The number of patients with each tumour type in the North and Franceschetti trials is a breakdown of the *total* number of patients in the trial, not only patients who were seizure-naïve.

b Results were reported separately for patients undergoing surgery for malignant glioma and for patients with metastases.

na = not applicable; nr = not reported; kps = Karnofsky performance status; mi = myocardial infarction.

**TABLE II:** Incidence of seizures in the randomized controlled trials included in the practice guideline

*Reference*	*Patients (*n*)*	*Treatment*	*Seizures (*n*)*	p *Value*	*Follow-up*
North *et al.*, 1983 7	42	Phenytoin	9	ns	12 Months
	39	Placebo	5		
Franceschetti *et al.*, 1990 8	41	Phenobarbital or phenytoin	6	nr	12 Months
	22	No treatment	7		
Glantz *et al.*, 1996 9	37	Divalproex sodium	13	0.3	Median: 7 months
	37	Placebo	9		
De Santis *et al.*, 2002 10	100	Phenytoin	13	>0.05	1 Week
	100	No treatment	11		
Forsyth *et al.*, 2003 11	46	Phenytoin	11	0.98	Median: 5.44 months
	54	No treatment	15		

ns = nonsignificant; nr = not reported.
